# Integration of HIV-Sexual Reproductive Health Services for Young People and the Barriers at Public Health Facilities in Mbarara Municipality, Southwestern Uganda: A Qualitative Assessment

**DOI:** 10.1155/2019/6725432

**Published:** 2019-04-10

**Authors:** Cecilia Akatukwasa, Francis Bajunirwe, Simpson Nuwamanya, Noel Kansime, Emmanuel Aheebwe, Imelda K. Tamwesigire

**Affiliations:** ^1^Infectious Diseases Research Collaboration, P.O. BOX 7475, Kampala, Uganda; ^2^Department of Community Health, Mbarara University of Science and Technology, P.O. BOX 1410, Mbarara, Uganda

## Abstract

**Background:**

Sexual and Reproductive Health (SRH) and HIV risk behaviors for young people are intertwined. This rationalizes the need for integration of HIV and SRH services within the health care system, especially in countries with high HIV burden. In this study, we explored the current status of HIV-SRH integration for young people and barriers of integration from different stakeholders at public health facilities in Mbarara Municipality, southwestern Uganda.

**Methods:**

We conducted an exploratory qualitative study at public health facilities in Mbarara district of southwestern Uganda. Data were collected among young people (n=48), health care providers (n=63), and key informants (n=11). We used in-depth interviews and focus group discussions to collect the data. Coding and analysis of qualitative data were done using Atlas.ti.

**Results:**

Overall there was no differentiation of HIV-SRH services between adults and young people. Integration of HIV-SRH services was reported at all facility levels; however, there was poor differentiation of services for the young persons and adults. Integrated HIV and SRH services for young people were acknowledged to improve access to information and risk perception, improve continuity of care, and reduce cost of services and would also lead to improved client-health worker relationships. The potential barriers to achieving HIV-SRH integration included individual provider characteristics like lack of training and attitudes, generic health system challenges like low staffing levels, poor infrastructure with lack of space and privacy to deliver these services. At the policy level vertical programing and unclear policies and guidelines were identified as challenges.

**Conclusion:**

Our study shows integration of HIV and SRH services exists in general but services for adults and young people are blended or poorly differentiated. Significant health system barriers need to be overcome to achieve differentiation of the services for young people and adults.

## 1. Introduction 

Young people are persons aged 15 to 24 years and are estimated to be almost two billion, with 90 percent of them living in low income countries where they form about one-third of the population [1]. This age group, especially the young women, is at high risk for HIV infection [2] because of exposure to high risk sexual behaviors such as no condom use, transactional sex and multiple sexual partnerships including early sexual debut [3] and disparate age sexual relations [4]. In sub-Saharan Africa, adolescent sexual and reproductive health (SRH) is often surrounded with myths and beliefs that may promote more risky behavior [5]. These increase the risk for other sexually transmitted infections and negative reproductive health outcomes which include unintended teenage pregnancy, a risk factor for poor maternal health outcomes [6].

Optimizing utilization of sexual and reproductive health services through integrating HIV and SRH services for this age group is a key step to mitigating the high risk sexual behaviors [7]. Although this group feels motivated to use family planning services and prevent HIV and unwanted pregnancies, they have limited access to information about where to access these services [8]. Majority of the developing countries are yet to meet the targets related to their commitments made to provide SRH services to the young persons [9]. In this age group, there is a high unmet need for contraceptive services yet also limited access to HIV/STI clinics and low uptake of HIV testing services [10].

Inadequate linkage of SRH services often leads to missed opportunities for addressing these unmet needs [7]. Overwhelming evidence indicates that integration of reproductive health services with HIV services would be one of the key steps to address these needs holistically [11]. The integration here refers to the process of bringing together SRH and rights, HIV interventions or operational programs to ensure access to comprehensive services in an efficient and effective manner [12].

Integrated SRH-HIV services are considered to have several benefits such as greater efficiency and cost effectiveness compared to stand-alone models, enhanced service access, and increased utilization of the separate service components [13, 14]. The integration increases client satisfaction and potential improvements in health outcomes due to the fact that a greater and higher quality of service access reduces the likelihood of missed opportunities and service related stigma [11, 15, 16].

Integration of HIV and SRH services has strong justification. About 80% of all new HIV infections are sexually transmitted [11]. Secondly, SRH and HIV/AIDS problems share the same root causes such as poverty, gender inequality, stigma and discrimination, and marginalization of vulnerable groups [17]. However these linkages are overshadowed by the predominant stand-alone models in terms of funding, programming, and service delivery [18]. Altogether, HIV-SRH integration has the potential to enhance SRH, contribute to reversal of the HIV/AIDS epidemic, and mitigate its impact.

In Uganda HIV and SRH services are among those that are largely provided on an out-patient basis. While integration of HIV-SRH services presents several advantages, the approach has not taken root to the level expected in Uganda and many countries in sub-Saharan Africa. And specifically, there is limited data on experiences of integration of HIV and SRH among young people. Where the process has been attempted, there is limited documentation of the successes, potential barriers and challenges. Therefore, the aim of this study was to assess the extent of integration of HIV and SRH services for young persons at public health facilities in western Uganda, exploring current practices and barriers to the integration.

## 2. Methods 

### 2.1. Setting

We conducted an exploratory qualitative study among healthcare workers and young people in public health facilities in the periurban location of Mbarara Municipality of South Western Uganda. Located 250km southwest of the capital, Kampala, the municipality has a population size of close to half a million residents. The study was conducted at health facilities comprised health center IIs, IIIs and IVs at parish, subcounty, and county level, respectively. We also conducted assessment at the district hospital. In Uganda, public health facilities are structured in eight service provision tiers. These include health center 1, which is a community health worker operating at the village level, health center II at the parish level, health center III at the subcounty level, health center IV at the county level, and the district hospitals. Beyond that we have the national and regional referral hospital. All these facilities offer SRH and HIV services.

### 2.2. Eligibility

The municipality has 9 public health facilities starting at level II and all of them were included in the study. All health workers at these facilities were selected to participate in the study, save for the hospital where we included health workers involved in HIV or SRH service provision. We also included in the study young people, aged 15-24 seeking health care at these facilities.

### 2.3. Data Collection

#### 2.3.1. Health Facility Observation

The service provision status and characteristics of the facilities were assessed using an observation checklist. This was done to assess how the different SRH and HIV services are provided at the different facilities especially for the young people. Client flow and availability of space among other characteristics of the facility were also determined.

#### 2.3.2. In-Depth Interviews

Individual interviews using a semistructured interview guide were conducted with health care workers at the public health facility. All health workers were eligible, but we excluded the in-charges in the different public health facilities as they engage more in administration. The data collection tool comprised open ended questions which elicited responses on providers' understanding of integration, providers' attitude towards integration, views on supporters and barriers to integration and experiences with providing HIV and SRH services to young people in their clinics. The interviews were audio recorded.

#### 2.3.3. Focus Group Discussions

We conducted six focus group discussions comprising 6-9 participants with a purposively selected group of young people. These were stratified in two subcategories, that is, in school and out of school. From each category a focus group was conducted with female unmarried and female married as well as male married and male unmarried young persons. The participants were identified at the facility within the municipality and invited to participate in the focus group discussions on a first come basis. The FGDs were conducted in* Runyankore, *the local dialect, and English depending on the subcategory. The FGDs were conducted to help to establish client experiences with services and opinions about integrated SRH-HIV services particularly the potential benefits of integration and barriers, their views on how services should be integrated and preferences. Before each FGD, the moderator introduced all the study participants and explained the general topic of discussion. The FGDs were recorded with an audio recorder and notes using an experienced field note taker were taken. After the FGD, the note taker and the moderator reviewed the written notes together while listening to the recording.

#### 2.3.4. Key Informant Interviews

Key informant interviews using an interview guide were conducted with eleven key informants. Open ended questions were asked to assess understanding of integration of HIV and SRH services, views on supports and barriers to integration, understanding of the policy environment and implications in relation to integration, and views on how HIV and SRH services should be integrated for young people. These interviews were also audio recorded.

### 2.4. Quality Assurance

The FGD guide, IDI guides, and key informant guide were pretested to check for accuracy and consistency and improve validity before the official data collection process. The FGD guide and consent form were translated from English to* Runyankore* for FGD participants who could not comprehend the English language. Two trained research assistants were recruited to conduct the data collection.

### 2.5. Data Analysis

The focus group discussions and key informant interviews were audio recorded and field notes were compiled during the data collection process. The recordings of the FGDs and key informant interviews were transcribed verbatim and reviewed in comparison to field notes. A thematic framework approach was used for data analysis. A code list was generated based on the objectives of the study. Coding and analysis were done using Atlas.ti software, where segments from the data were copied and assigned to the generated codes. Texts were coded and clustered along emergent themes in the data and these were later organized according participant's understanding of integrated HIV and SRH services, importance of provision of integrated HIV and SRH services to young people and factors affecting the provision of integrated HIV and SRH services to young people (supports and barriers). The qualitative data were presented in narrative form.

### 2.6. Ethical Considerations

Research and ethical clearance were obtained from the Research and Ethics Committee (REC) of Mbarara University of Science and Technology (IRB Number: 08/06-13). Informed consent was obtained from all the study participants. We ensured privacy and confidentiality during the data collection by interviewing participants in closed rooms and used codes to identify participants. Only the participants' identification number was included on the transcripts and questionnaires. All data were stored in password protected computer files accessible only to the study team.

## 3. Results 

### 3.1. Characteristics of Respondents

We conducted facility observation in all 9 public health facilities of Mbarara Municipality and these comprised 4 health center IIs, 3 health center IIIs, 1 health center IV, and the district hospital. Four of the 9 facilities were urban and the remaining 5 were periurban in location. The proportion of young people attending the health facilities as fraction of all persons attending the health facilities was 36.5% at HC IIs, 45.3% at HC IIIs, 42.1% at HC IVs, and hospital 40.7% at the hospital. We conducted six focus group discussions (FGDs) with a purposively selected group of young people. Each FGD consisted of approximately 6-9 participants who were identified through the facility. Altogether, a total of 48 young people with a mean age of 20.83 years participated in the FGDs and the details of the composition of the FGDs are shown in Table 1.

We conducted individual interviews with 63 service providers spread across all the 9 public health facilities in Mbarara Municipality. Eleven key informant interviews were conducted and these included 6 interviews with in-charges of the selected public facilities, 1 with a district official, and 4 with program managers of selected NGOs operating within the municipality. The key informants had a mean age of 34 years and the median years of experience were 5 years.

### 3.2. Characteristics of the Study Health Facilities

The study was conducted at 9 public health facilities and the characteristics are shown in Table 2. For a period of six months, young clients ranged from 36.5% at HC II to 45.3% at health center IIIs. Female clients generally outnumbered the male clients.

### 3.3. Differentiation of HIV-SRH Services for Young People from Those of Adults

In relation to the services provided, all the nine facilities were able to provide HIV and SRH services either under one roof or within separate buildings within the health facility location or compound.

A provider at one health facility mentioned that

These days at the different health facilities at the different levels, you find they are almost in position to provide a comprehensive package of HIV and SRH services. They can do HIV testing, they can do family planning, they can do maternal care, and they can do screening for reproductive health cancers** (Female Clinical Officer #1)**.

We observed in three-quarters (75%) of the nine public health facilities in the municipality that clients formed a single queue to see one provider regardless of their age. This was observed in health centers IIs and IIIs for all services except for antenatal care and immunization clients. At the health center IV, HIV services were provided in a separate building from the main facility buildings. The services were delivered through a separate program area. Family planning and maternal and child health (MCH) services were also provided in separate rooms.

At the hospital, clients queued separately for specific services, since the hospital has separate departments for the different services that are required by the clients, specifically HIV treatment and care services. Maternal child health (MCH) and HIV testing services were all provided in separate department buildings. Saving for one HIV clinic at the hospital, we observed that young people at all the other health facilities accessed services alongside the adult clients. It was only in the district hospital where a separate queue was observed for adolescent HIV clients who attended to in the pediatric section. This assertion was established by one provider.

Government health facilities don't look at whether you are a young person or whether you are an adult. Regardless of what age you are; they look at people seeking services through the main stream. They serve you as you come. So the young people join the line with adults** (Female Nursing Officer #1)**.

Another provider in a health center III gave the same account that there was no special consideration for the young people seeking services at most government health facilities.

We don't have a specific time or room for them; we give services according to first come first serve but usually the youth have a characteristic of coming at awkward hours lunch time or when we are about to close so we wait and offer them the services because we know that's how they are** (Female Clinical Officer #1)**.

### 3.4. Current Practices of HIV-SRH Service Integration for Young People

The records of the family planning register, HIV counseling and testing registers and the out-patient department register showed that HIV counseling and testing (HCT) were integrated into family planning services and this was routine. According to the family planning register, the majority of the clients who sought family planning services were offered HCT services as well during the same visit and details for these services are shown in Table 3. In this table, we assessed the proportion of young family planning clients that received HCT. Data collected over a 3-month period show that at least 80% of the young family planning clients had received HCT at all health facilities saving for the district hospital. However, there was no record of reproductive health services that were offered to young people in the HCT register.

The providers also revealed that besides the routine family planning-HCT service integration, they also offered integrated HIV-SRH services to their clients in the same visit though not on a routine basis. For example HCT for clients seeking care for a sexually transmitted infection (STI) was done when time allowed. This was mainly observed at the health center IIIs.

“What we do if someone comes with an STI we have to test for HIV, it is not mandatory but given the information most of them they do accept and we do the test for HIV”** (Female Clinical Officer #1)**.

On the other hand the young people recounted that in some instances when they came to receive particular services not necessarily HIV or SRH services, they had the opportunity to access other services which were mainly information services.

They teach us. At some health facilities when you go to receive services like to test for pregnancy and you get there and find a group being taught and you join them, you find them discussing about STIs and how to protect ourselves as youth from HIV** (Female out-of-school married FGD #1)**.

Other young people also recounted some of their experiences at the health facilities regarding integration.

Participant 1: I went and tested for HIV and then I was given information about STDs.

Participant 2: I went to check for HIV and they gave us condoms to use and distribute to others.

Participant 3: I went for circumcision and I was given condoms and a book.

**(Male in-school unmarried FGD #2)**

### 3.5. Perceived Benefits of HIV-SRH Integration for Young People

Participants' perspectives on the benefits of provision of integrated HIV and SRH services for young people were explored further and categorized in the following thematic areas: cost effectiveness in terms of time and money for the young people, privacy and confidentiality, continuity of care, improved relationship between the provider and young client, increased knowledge and risk perception.

#### 3.5.1. Cost of Services

From a client's perspective, young people reported that integration would help them cut costs in terms of transport costs and time spent trying to receive the required services.

If you came to this facility and one service is provided you will have to go to another facility to seek for another. Maybe you want to do syphilis test or gonorrhea; you will go to another facility where it is done. So you can see how much money you will have spent moving from one facility to another. How much money, how much time will you have saved if you get all your services from here?** (Male in-school unmarried FGD #2)**.

Similarly the providers recounted that with a one stop facility, young people would be able to seek a variety of services in the shortest time possible given their nature of having no time for their health.

Young people don't have a lot of time for health; health is not like a priority to most of them and so if they have to go looking for services in different places it is a waste of time. So having an integrated one stop center you catch them [snaps fingers] they come for 2 minutes you catch them there, you give them everything, give them all the information [snaps fingers] and you save on time** (Female Enrolled Nurse #1)**.

### 3.6. Building Trust and Improving Client-Health Worker Relationships

Many young people emphasized that integration of HIV and SRH services for young people would help them build trust with their service providers and also promote or create a positive relationship between the service providers and the young clients. This was also revealed by the young people.

…if one provider is handling all your problems you feel attached to him and you feel open to him. You tell him each and every problem of yours so that he gives a solution in time and then you go on** (Male out-of-school unmarried FGD #3)**.

A key informant also expressed a similar sentiment on the client-provider relationship which would be enhanced by integration of HIV and SRH services.

These young people need an attitude that is welcoming, an attitude that is friendly that is respectful of them even as young person. When we have those kinds of services integrated, we would have the young people confiding in the health workers, because they are able to dig out all their problems regarding their sexuality** (Female Program Manager #1)**.

#### 3.6.1. Continuity of Care

HIV-SRH integration prevents clients from dropping out of services because they cannot access the required services in one place.

When you go let's say you have an STI then maybe they tell you to test for HIV and so on maybe they refer you to another place for such services and in that process of shifting from this place to another people tend to drop, “Ok fine I will go there” but in the end they don't go there** (Male in-school unmarried FGD #2)**.

It was emphasized that given the nature of youth in relation to their poor health seeking behavior, having to access different services in different places would cause them drop out; however, with integration, young people can be captured and are able to access a variety of services at a go in the least time possible.

Young people don't have a lot of time for health; health is not like a priority to most of them and so if they have to go looking for services in different places it is a waste of time. So having an integrated one stop center you catch them [snaps fingers] they come for 2 minutes you catch them there, you give them everything, give them all the information [snaps fingers] and you save on time.** (Female Enrolled Nurse #1)**.

#### 3.6.2. Access to Information and Risk Perception

Integration of HIV and SRH services for young people would serve as a channel through which information on HIV and SRH issues would be conveyed to the young people; consequently they become well informed in a holistic manner because they are able to access an all-inclusive set of information on their sexuality from different perspectives.

So the most important benefits that will come out of integrating the services are that at least you have an informed kind of person of all the ailments that come from either way, from the HIV/ AIDS perspective and the reproductive health perspective. So what we would want to benefit out of it is that seeing an informed person, or seeing that we can offer a full package to someone not leaving other risk factors pending just because maybe we are not concerned about it** (Female Nursing Officer #1**).

The young people believed that with integration their perception of other risks regarding their sexuality besides HIV would improve.

Currently I think integration would be good for us now our fear has mainly been focused on pregnancy. But now we can get knowledge about HIV prevention or pregnancy; two things, because all of them are caused by unprotected sex** (Female in-school unmarried FGD #4)**.

It was also mentioned that integration would be the best channel through which information on HIV and reproductive health would be relayed to the young people since the other sources like the parents and peers may not be reliable in terms of knowledge and availability.

### 3.7. Factors Influencing HIV-SRH Service Integration for Young People

#### 3.7.1. Provider Level Factors

*Sociodemographic Characteristics of Key Informants*.  Table 4 is a summary of the characteristics of the providers that participated in the study. Majority were female (55%) and the majority (92.31%) have spent less than ten years at the health facility. Median age was 34 years, and majority (44.2%) of the providers was aged between 31-40 years.

During the in-depth interviews, it was not clear whether these characteristics affected HIV-SRH integration for young people. However some young people expressed concern over receiving SRH and HIV services in facilities that had older providers.

I do not know why but in government hospitals we find very old providers and these ones have a negative attitude towards us the youth. I find it difficult if I want to test for HIV and I find an older woman what do you think she will think about me, even when she does not say a thing, that is what you will take in your heart, so when we find them we just go back** (Female out-of-school unmarried FGD #5)**.

*Provider Competence and Training*. Service providers were asked to rate themselves on how competent they felt to be able to provide integrated HIV and SRH services to young people. The majority (60%) of the providers stated that they did not consider themselves competent enough and thus required some training and practice to be able to offer integrated HIV-SRH services to young people while only 40% of the service providers felt that they were competent enough to offer these services to young people. This deficiency in competence was also recounted by the key informants:

The other issue we have is the staff competence. Providers are not well skilled to serve young people, and to provide the required sexual reproductive health services in one visit** (Male Program Manager #2)**.

Most providers reported that they lacked adequate skills and training was expressed as a concern for integration of HIV and SRH services for young people.

Training is also another challenge especially for health workers that deal with young people. No one has been trained here in handling youth a part from the in-charge. He is the one who knows because he goes for some talks. He has been trained on youth counseling, youth testing. The rest of us use what we learnt from school** (Female Nursing Officer #2)**.

It was also mentioned that a lot of training was inclined mostly towards HIV services and the reproductive health services had not been well considered.

We also have training, we have been trained on HIV at least every one here can handle HIV related issues, everyone can handle an HIV patient, anyone can provide services you know, it should also be like that because you know the problems of reproductive health are the ones which are leading to HIV. So we are only confronting HIV but we are not seeing that the youth have other reproductive health problems** (Female Clinical Officer #1)**.

#### 3.7.2. Service Related Factors

*Equipment and Supplies*. Stock outs of drugs and supplies were also noted as a challenge. At some health centers most clients were being turned away because there were no HIV testing kits. The ones available were for pregnant mothers. So clients that sought HCT were turned away and were not offered any other available services.

Whenever we come here and say we would like to test they tell us there are no testing kits come back another time, we come back and it is the same story** (Male unmarried out-of-school FGD #3)**.

…for the past six months there has been a very big crisis in terms of condom shortage and we know that the biggest proportion of sexually active people is the young people. So in the absence of condoms, what do you expect them to be using** (Male Program Manager #1)**.

#### 3.7.3. Health System Level Factors

During observation, focus group discussions and key informant interviews, the study participants highlighted comparable challenges to integration such as staffing levels, client loads, stock outs and limited working space among other challenges.

*Staffing Levels*. Most facilities reported having fewer providers than the stipulated number of providers expected at each health facility. The effect of the staff shortage is that there would not be providers designated to provide services or prioritize special populations such as the young people. This was recounted by different study participants.

In a setting where you have very few health workers or service providers for the population all the providers are engaged at the facility and because they are engaged, there are no special priorities, attention given to particular groups of the population like young people is limited** (Female Nursing Officer #1)**.

More so besides the shortage of staff, most providers said that they had big client loads. However this was only vivid in the higher facility levels, that is, the health center IV and hospital. It was observed that in the lower level facilities clients tended to see one provider for all their ailments unlike in the higher level facilities (HCIV and hospital) where services were running parallel in the different departments. However, it was also reported that in the presence of one provider handling all clients certain needs of the young person would be left out.

If we are to provide integrated HIV and SRH services to young people we need providers to handle all these aspects yet the few available providers do not have all the skills nor the time to handle all the needs of the young person** (Female Clinical Officer #1)**.

*Infrastructure*. The following infrastructural elements were observed, that is, auditory and visual privacy of consultation rooms, space in the waiting area, and working space for the providers. There was audio and visual privacy for consultation rooms in 6 of the 9 health facilities. For the other three facilities it was easy to listen to what the patient and provider were discussing since the window of the consultation room was facing the waiting area. The VCT clinic at the upper level facilities lacked privacy as clients had to line up outside the clinic to access services.

Sometimes the youth are not comfortable seeking services here who are not maybe because of their age because the facility lacks a private space for them to wait** (Female Clinical Officer #1)**.

The young people also expressed a similar concern.

When we go to a government health facility we have to line up with everyone yet we fear because if you have an STD or you want to get HIV test, you cannot stay there because the friends of your parents or relatives are watching and they always want to know what you are doing there. So you start to wonder “how will I go to this facility where I'm likely to meet the same people** (Female out-of-school unmarried FGD #5)**.

From observation, majority of the facilities had space in the waiting area and 5 of the 9 facilities had in-built seats for clients. However, space in the waiting area was available depending on the day of the week. For example on some heavy clinic days like immunization and antenatal care days for some clinics, patients were seen waiting in the compound because all the seats in the waiting area were taken. This was seen in 7 of the 8 facilities. At the hospital, which had services offered within several departments, there was variation in availability of space varied between departments. For example there was sufficient space at the out-patient department; however, at the MCH department and HCT space was always insufficient.

Working space for the providers was also observed to be a challenge. We observed in some facilities that services had to be shared rather inconveniently. For instance, in one health center III, the laboratory doubled as the consultation room. We also observed at 2 facilities that the consultation rooms doubled as storage rooms. This was also highlighted by several participants as deterrent to integration. The participants felt that there was not sufficient space to provide certain services.

…we also work within a very limited space, if you look at the facilities that we have, Health center II. Health center III even health center IV they work in very small rooms. Now you would want to have a waiting space for young people, you would want to have an examination room where young people are examined; you would want to have a treatment room; that cannot be possible** (Female Nursing Officer #2)**.

You find the structures in place can accommodate some services and cannot accommodate extra services, so they decide to concentrate on those services where they think they can accommodate** (Male Program Manager #2)**.

#### 3.7.4. Policy Level Factors

Policy related factors that were highlighted included availability of policies and guidelines, challenges to implementation of these policies such as continuously implementing vertical programs and services and priorities of funding sources.

*Policies and Guidelines*. Most respondents reported policy constraints as one of the common obstacles for clinics to overcome during integration of HIV and SRH services for the young people. According to most informants, it was reported that there were policies in place supporting provision of services to the young people in a more youth friendly and integrated manner; however, this was not systematic and implementation of these policies at service delivery level was still a challenge.

There is a policy on adolescent reproductive health; there is also another policy on provision of integrated youth friendly… reproductive health services, but the implementation of the policy is what is lacking** (Female Nursing Officer #2)**.

It was also stated that despite having policies in place to support provision of SRH and HIV services to youth in a more integrated manner, young people remained being continuously served in the mainstream with the rest of the population.

As I speak right now the government health facilities do not look at whether you are young person or whether you are an adult. Regardless of what age you are, they look at people seeking services through the main stream. They serve you as you come. So the young people can join the line with adults** (Female Enrolled Nurse #1)**.

*Vertical Funding*. Another health system challenge identified was funding. It was revealed that there was a limitation in terms of funding simply because some of the facility activities are driven by implementing partners, implying that the different funding sources give priority to activities of their interest.

I think another limitation is funding, a lot of our services are donor driven. So you find that the health facilities have a comprehensive set of services for the young girls or the boys and even the women, you find there is a project, a project funded by a certain funder and another project funded by another one. Each is offered as a separate entity depending on the terms of the funder. And also when you go to another health facility which doesn't have the privilege of having these kinds of projects you find some of the services are not there because probably the government cannot support the availability of all those clinical services in one place** (Female Program Manager #1)**.

For example, it was mentioned that many funding agencies were focusing a lot on HIV service provision and a few were focusing on SRH, and this would have implications on integration.

It's been proven that some areas have not been given priority in terms of funding or they have been offered in isolation. From statistics you will see that not only are we scaling down the AIDS prevalence but instead it is increasing, because other risk factors have been handled separately. For example different agencies focus a lot on HIV/AIDs services and not reproductive health** (Male Program Manager #2)**.

This was clearly observed in all facilities where HIV services are funded and provided separately from other services under a separate program. Notably, in two of the nine facilities, HIV services were offered in a separate building under the management of a different program funded by a certain agency.

In addition to that, even in the presence of donor driven activities, most providers noted that there were still insufficient funds to facilitate activities relating to HIV and SRH services for the young people such as the professionals to work with the young people, and the materials to offer the services.

In most cases they tell you funds, that yeah we would wish to do this activity but we have no funds to do it. Because funds control other factors, the professionals to work on it, the materials to use for example if you are to test AIDS you need kits, you need maybe professionals and they need to be paid and so on. So in most cases there is a challenge of funds** (Female Nursing Officer #3)**.

## 4. Discussion 

This study provides an exploratory qualitative analysis of HIV-SRH integration for young people in a setting where a national HIV and SRH integration and linkages strategy was developed and endorsed in 2012 [12]. While several other studies document HIV-SRH integration practices for the general population [19, 20], this study extends previous research by providing novel findings on the status of HIV-SRH integration practices for young people aged 15-24 years in Uganda. Our data show presence of HIV-SRH integration though not explicitly structured and differentiated to suit the young people who are severally reported as a high risk group for HIV infection and other SRH risks compared to other age groups. Overall, the young people, service providers, and health managers in this study expressed favorable attitudes towards integration of HIV and SRH services for young people as it fosters increased service access and utilization among other benefits. However several health system barriers were reported to potentially inhibit full realization of service integration.

Two distinct forms of integration reported in this study comprise the facility level and the service delivery level. At the facility level, all HIV and SRH services existed within the same facility location regardless of the health facility. However this did not guarantee that the clients received an integrated package of these available services in a single visit. At the service delivery level, clients could access all available services in a single clinic visit irrespective of whether services were offered by one provider or different providers through an internal referral. This was specific for provision of HCT within family planning services. The service delivery mode of integration further generates distinct forms of integration depending on the facility level. In the lower health facilities, namely, health center II and III, SRH-HIV services were most likely to be offered by one provider while the higher level facilities (HCIV and hospital), services such as MCH, antiretroviral and STI treatment, were provided in dedicated separate departments or rooms which required movement of the client from one department to another. Continuity of care at higher facility levels was more likely to be compromised due to high chances of drop out in between referrals. This would affect the young people especially whose health seeking behavior is poor. The lower health facilities are more likely to provide a more explicit model of integration compared to the higher level facilities as clients formed a single queue to see one provider and specialization at this level would be diminished [21].

Many vertical programs at higher level facilities in themselves can integrate services, for example, family planning counseling and reproductive health counseling within the ART clinics. HIV screening could take place within the STI clinics. Condom availability was cross cutting across the facility levels though, in some instances, condom supplies dwindled. These variations of integration imply that integration can be achieved differently for every facility level [22]. However, there is a need to conduct more research to identify the best integration models as these are likely to be different from one setting to another [22].

Nevertheless integrating HIV and SRH services for young people was considered beneficial. Our study shows that integrated services promote continuity of care and improved client-provider relationships. Our data suggest an increased risk perception and increased HIV-SRH service demand and utilization among young people where there is integration. Studies have shown that integrating HIV testing into family planning services for young people would scale up their uptake of HIV testing services which are underutilized by the young people [23]. This would also serve as an entry point for HIV prevention and timely linkage into HIV care [24]. However health system gaps in addressing the needs of young people have decelerated this kind of integration. Noteworthy, most family planning services in Uganda are mainly inclined towards the females implying missed opportunities for the males to access testing services. Studies on HIV-family planning integration have reported that men comprised less than 5 % of patients seeking HIV testing services [24]. This calls for a need to scale up the utilization of family planning services for males through leveraging male participation in family planning services. Other approaches of integration reported include HIV testing within the STI clinics. Studies have shown that HIV testing within STI clinics led to higher chances of acceptance of HIV testing especially among males [25].

Although our data show significant integration of SRH and HIV services, there was profound lack of differentiation of services for young people. The young people in our study expressed concern they were uncomfortable lining up with adults to access services that some people perceived as services for adults. They were uncomfortable they might encounter family members who might judge them. Stigma has potential to reduce uptake of SRH-HIV services, even when they are well integrated as long as there is poor differentiation of services for young persons and adults. Second, the data show integration of HIV into SRH and less SRH into HIV. For adequate integration to take root, one might expect to see a bidirectional approach in order to meet the needs of young people.

Our data indicate that HIV-SRH integration is curtailed by number of factors. Availability of working space, competent staff, low staffing levels, and insufficient equipment and supplies were found to be among the factors influencing the integration of HIV-SRH services for young people. Similar findings are highlighted in previous studies [19, 21, 26]. Integration of HIV and SRH services especially at the provider level in the lower health facilities would require the provider to have skills in all aspects of reproductive and SRH services, assessing risk as well as dealing with young people. However, over 60% of the providers in our study expressed concern over their competence to effectively offer youth friendly services. Even more, these health care workers are already burdened with heavy workloads which limit their ability to holistically address the health needs of the young people. The heavy workload is accompanied by a heavy client load which in the long run increases the waiting times of clients. Along with this, we observed that majority of health facilities lacked audio visual privacy as well as convenient working hours to serve the young people. This combination of factors implies major deficiency for these facilities to address the needs of young people.

One of the major strengths of this study is that we assess integration of SRH-HIV services with a focus on young people. Majority of studies have examined integration in general. Second we triangulate our data across the participants and the data collection methods. Our study has some weaknesses. First, we conducted the study at public health facilities in an urban setting. Although this was deliberate, the findings may not be representative of rural and private health facilities. Second, we did not collect data on client-provider interactions between the young people and the providers. These data would have contributed significantly to the study findings by clearly highlighting the key integration experiences. Further research investigating best integration models for young people with data on client-provider interactions is needed.

In conclusion, access to HIV and SRH services for young people remains a public health challenge especially in high HIV prevalence settings. There is urgent need to refocus efforts to promote integrated services among the young people at the health facility and policy level. Integration of HIV and SRH services will help to increase service utilization, promote HIV testing, and improve risk reduction behavior among young people. There is also a need to further understand the contexts in which integration takes place especially at the health system where many generic challenges were reported.

## Figures and Tables

**Table 1:** The composition of focus group discussions for young people at health facilities, Mbarara Municipality.

FGD No.	Gender	Marital status	Mean age	Schooling status	No. of participants
1.	Female	Married	22	Out	6
2.	Male	Married	23	Out	8
3.	Female	Single	20	In	9
4.	Male	Single	19	In	9
5.	Male	Single	21	Out	7
6.	Female	Single	20	Out	9

**Table 2:** Characteristics of study health facilities in Mbarara Municipality.

Facility level	HCII (n=4)	HCIII (n=3)	HCIV (n=1)	Hospital (n=1)
*Location*				
Urban	1	1	1	1
Peri-urban	3	2	-	-

*Cadre*	.		.	
Medical officers	NIL	NIL	4	6
Clinical officers	NIL	6	4	3
Nurses	12	13	7	11
Midwives	6	6	7	8
Counselors	-	-	2	6

*Total client loads*	312	306	439	825

*Young client loads for six *	114 (36.5%)	139 (45.3%)	185 (42.1%)	336 (40.7%)
*months at OPD*				
Males	45 (39.5%)	63 (45.3%)	83 (44.9%)	144 (42.9%)
Females	69 (60.5%)	76 (54.7%)	102 (55.1%)	192 (57.1%)

**Table 3:** Proportion of young family planning clients who received HIV counseling and testing between June and August 2016 in Mbarara Municipality.

Facility Level	HCII	HCIII	HCIV	Hospital
No. of FP clients.	30	33	117	235
No. of young FP clients n (%).	10 (33.3)	18 (54.5)	51 (43.6)	78 (33.2)
Number of young FP clients who received HCT n (%).	08 (80.0)	15 (83.0)	45 (88.0)	54 (69.0)

**Table 4:** Characteristics of key informants at health facilities and programs in Mbarara Municipality.

Category	n=11
*Cadre:*	
Program managers	4 (36%)
Facility in-charges	6 (55%)
District officials	1 (09%)

*Gender:*	
Male	5 (45 %)
Female	6 (55 %)

Age (years)	34 ( 28-43)
Median (range)	

Years of experience	5 (0.5, 22)
Median (range)	

## Data Availability

All data used in this analysis are available with approval from relevant local authorities.

## Abbreviations

AIDS: Acquired Immunodeficiency Syndrome
HC: Health Center
HIV: Human Immunodeficiency Virus
ICPD: The International Conference on Population Development
SRH: Sexual and Reproductive Health
UNFPA: United Nations Population Fund
YPLHIV: Young People Living with HIV.
